# Study of Interactions between Titanium Dioxide Coating and Wood Cell Wall Ultrastructure

**DOI:** 10.3390/nano12152678

**Published:** 2022-08-04

**Authors:** Petr Svora, Sylwia Svorová Pawełkowicz, Petra Ecorchard, Jiří Plocek, Alena Schieberová, Zdeněk Prošek, Petr Ptáček, Jan Pošta, Piotr Targowski, Petr Kuklík, Ivo Jakubec

**Affiliations:** 1 University Centre for Energy Efficient Buildings, Czech Technical University in Prague, 273 43 Buštěhrad, Czech Republic; 2 Institute of Physics of the Czech Academy of Sciences, Na Slovance 1999/2, 182 21 Prague, Czech Republic; 3 Institute of Inorganic Chemistry of the Czech Academy of Sciences, 250 68 Husinec-Řež, Czech Republic; 4 Department of Chemistry and Chemical Technologies, Faculty of Wood Sciences and Technology, Technical University in Zvolen, T.G. Masaryka 24, 960 53 Zvolen, Slovakia; 5 Institute of Physics, Faculty of Physics, Astronomy and Informatics, Nicolaus Copernicus University in Toruń, Grudziądzka 5, 87-100 Torun, Poland

**Keywords:** titanium dioxide (TiO_2_), protective layer, photodegradation, beech wood (*Fagus sylvatica*), pine wood (*Pinus sylvestris*), wood cell ultrastructure, wood preservation

## Abstract

Titanium dioxide (TiO_2_) is used as a UV light absorber to protect wood matter from photodegradation. In this paper, interactions between wood and TiO_2_ coating are studied, and the efficiency of the coating is evaluated. For the experiments, two wood species were chosen: beech (*Fagus sylvatica*) and pine (*Pinus sylvestris*). Molecular and physical modifications in coated and uncoated wood exposed to UV radiation were investigated with Fourier transform infrared spectroscopy with attenuated total reflectance (FTIR-ATR) and transmission electron microscopy (TEM). UV-VIS spectroscopy was used to describe the absorption of UV light by the TiO_2_ planar particles chosen for the experiment. It was demonstrated that TiO_2_ coating protects wood against photodegradation to a limited extent. TEM micrographs showed fissures in the wood matter around clusters of TiO_2_ particles in beech wood.

## 1. Introduction

Chemically, wood mainly consists of polysaccharides (cellulose and hemicelluloses (xylans, mannans, β-glucans, and xyloglucans)), and lignin—an organic polymer built from three basic monomers, namely guaiacyl, syringyl, and p-hydroxyphenyl subunits [1]. Minor constituents of wood are extractives (such as resins, terpenes, gums, tannins, fatty acids, etc.) and inorganic substances (silica sand, druses, raphides, etc.) [2]. These chemical compounds are distributed in different parts of wood cells. Chemical composition of the wood cell has an impact on the fiber properties. For instance, lignin is responsible for compressive strength properties, hemicelluloses for the dimensional stability of wood cell walls, and cellulose for the wood’s stiffness [1].

All wood is subject to deterioration processes triggered by a wide variety of factors. Biological decay of wood is caused by fungi and bacteria, as well as insects. Physical factors, such as temperature, UV light, and relative humidity, contribute to the weathering of wood and affect wood properties.

Ultraviolet (UV) radiation, due to the high energy of its photons, breaks the chemical bonds of the organic compounds of wood matter. It is estimated that UV radiation penetrates through wood surface up to 70 µm, while photooxidation reactions may occur at depths above 200 µm [3]. UV light-induced degradation (photodegradation) of the major constituents of wood is responsible for the decrease in mechanical properties of wood and color change. With time, yellowish untreated timber turns brown because of decomposition of its surface. If exposed to the influence of fluids, such as rain, decomposition products are washed out, and the surface turns gray, while the performance of a wooden construction decreases. 

As wood is one of the oldest building materials, a vast range of protective products has been developed through the centuries. These are mostly various preservatives for impregnation, but there is also a large group of coatings [4,5]. Obviously, wood-related nanotechnology is currently in full swing [6]. Titanium dioxide (TiO_2_) [7,8,9,10,11], along with other nanosized metal and metal oxide particles (copper-based nanoparticles [10,12], zinc borate, zinc oxide [8,13], and silver [10,12]) are applied in coatings because of their antimicrobial properties. For this purpose, photoactive crystalline forms of TiO_2_ are used—pure anatase or anatase mixed with rutile, i.e., P25 [7], other commercial TiO_2_ nanoparticles (NPs) [8], or doped TiO_2_ NPs [10,11]. The role of the dopant is to enhance the antimicrobial effect of TiO_2_ NP and shift the absorption of TiO_2_ NP from UV toward VIS radiation [11]. 

Titanium dioxide has also been discussed in the context of superhydrophobic [11,14,15] and, usually simultaneously, UV protective surfaces [16,17,18,19,20,21,22,23,24,25,26]. The use of TiO_2_ as a UV light absorber to protect wood matter from photodegradation lies at the core of this research. The form of TiO_2_ NPs most frequently used in UV protective coatings is rutile [16,17,18,21,26] because it has lower photocatalytic properties than anatase. The latter is only rarely reported [18,23]. Although this is not clearly stated, based on previous experiments [27], we can assume that amorphous TiO_2_ NPs were studied by Rassam et al. [19] as the annealing temperature was too low (120–150 °C) to obtain crystalline structures. For coating stability, photocatalytic properties need to be suppressed so as to avoid interactions between TiO_2_ and the binder [17], or TiO_2_ and the wood matter [21]. Zheng et al. [21] reported interactions between rutile particles and wood caused by photocatalytic degradation of wood components. To suppress photocatalysis, insulating layers of silica or alumina were introduced to coat TiO_2_ particles [17,26], or multilayer coating systems were developed [22,28]. UV protective coatings usually consist mainly of TiO_2_ NPs, a binder, and various coalescing agents, e.g., organic surface active agents [16] or Texanol [26], to prevent nanoparticles from aggregating and ensure transparency of the layer. The binders used are usually acrylic copolymers [16,17,26]. In certain experimental studies, UV stabilizers, such as benzotriazoles and triazines, VIS radiation stabilizers—hindered amine light stabilizers (HALS)—Tinuvin [22,23,28], were used along with TiO_2_ NPs and, additionally, with ZnO NPs [22,23]. Different shapes of nanoparticles are reported in the literature describing experiments with UV protective layers: non-spherical [17] and spherical [18,19,20,24]. This research investigates the properties of planar TiO_2_ particles. The coating, apart from traditional brushing [22] or spraying [23], may be deposited in different forms: the sol-gel deposition process with dip-coating [19,20], hydrothermal methods [18], or the use of plasma [24]. Due to elevated temperatures, the last two methods may lead to the formation of a chemical hydrogen bonding between wood surface and TiO_2_ NPs [18,24], otherwise TiO_2_ is reported to be inert, and only physical interactions between wood and TiO_2_ layers may be expected because of UV energy absorption.

While most of the studies on TiO_2_ as a wood preservative show the results of color change [8,9,15,22,23], mechanical properties [15,26], or microbiological tests [7,8,9,15], little is known about microscopic and molecular changes occurring in wood under the influence of powerful nanoparticles. Infrared spectroscopy (FTIR-ATR) has been used in only a few studies [8,9,15,19,24,26]. To the best of the authors’ knowledge, none of the studies on TiO_2_ and wood used transmission electron microscopy (TEM) to observe changes on the level of wood ultrastructure. Usually, TEM studies addressed wood morphology or wood biodegradation [29,30,31]. 

**Figure 1:**
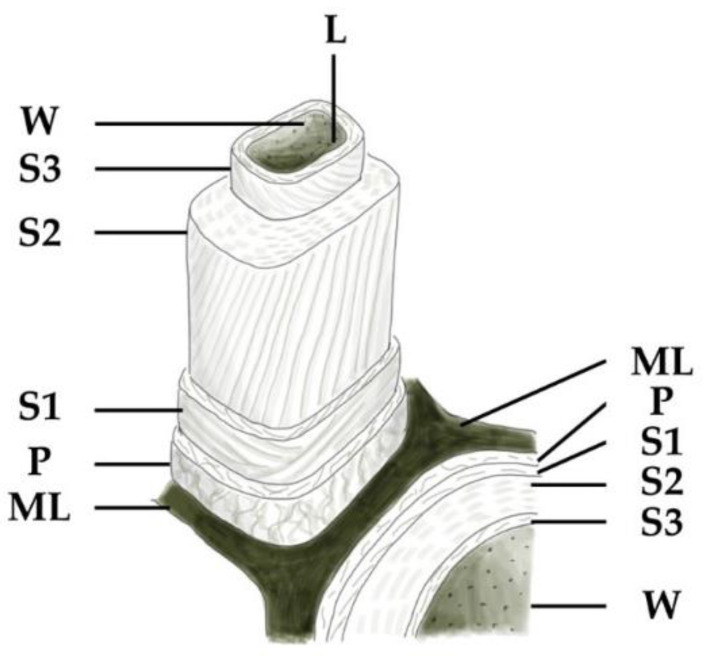
Model of a wood cell. Drawing by S. Svorová Pawełkowicz.

Chemical changes in wood composition, caused by degradation, are reflected in wood cells. The ultrastructure of wood has been studied extensively since the 1930s, using various analytical techniques (optical and electron microscopy, X-ray diffraction, infrared and Raman spectroscopy, and others) [1]. The general organization of a wood cell is well-known, and the nomenclature established is presented in Figure 1. The wood cell wall consists of: ML—middle lamella, P—primary wall, secondary walls, which are built from layers—S1 (outer), S2 (middle), S3 (inner), W or WL—warty lamella, L—lumen (empty part inside the fiber). This research uses transmission electron microscopy (TEM) to investigate the changes occurring under the influence of UV radiation in the wood cell ultrastructure of pine and beech wood coated and not coated with TiO_2_. 

The aim of this paper is to study interactions between planar titanium dioxide particles and wood matter. The paper focuses on describing the phenomena occurring as the UV energy is absorbed by a TiO_2_ layer built from amorphous, so, by definition, non-photocatalytic TiO_2_ particles. The key issue is whether this energy can affect the morphology and molecular composition of wood’s surface. For the study, beech wood (a representative of broadleaved trees) and pine wood (a representative of conifer trees) were chosen, as both are easily degradable by UV light [32]. Beech and pine sapwood are woods most commonly used for degradation tests because of their low resistance to biological decay (see standards EN 113 [33], EN 839 [34], and EN 252 [35]). Furthermore, pine sapwood is recommended in standards EN 927-3 [36] and EN 927-6 [37] for testing the durability of coating materials and coating systems for exterior constructions. Wood specimens have been coated with planar particles of TiO_2_ mixed with water, acrylic resin, and water glass. The use of binders complicated the system but at the same time made the study more realistic as, in the long run, nanoparticles will not stick to a wood surface exposed to external conditions with van der Waals forces only. The choice of acrylic resin was dictated by its popularity and nontoxicity, and its mechanical properties (elasticity) [26]. Nevertheless, an additional reaction between organic acrylic matrix and TiO_2_ was expected, as reported in the literature [6,26,38]. That is why we looked for an inorganic matrix, such as water glass. Wood specimens were submitted to artificial aging simulating environmental conditions. The absorbance of UV radiation of TiO_2_ particles was measured by UV-VIS spectroscopy. X-ray fluorescence (XRF) mapping was helpful in locating the proper place for extracting samples for further measurement from the wood specimens. Irradiated and reference wood specimens were inspected with transmission electron microscopy (TEM) to describe changes in the wood’s ultrastructure, and with Fourier transform infrared spectroscopy (FTIR) to describe the chemical changes triggered by the interaction of TiO_2_ and UV light. The efficiency of TiO_2_ coating has been proved, albeit to a limited extent. Fissures in the wood matter of beech wood were observed around clusters of TiO_2_ particles. 

## 2. Materials and Methods

### 2.1. Materials

#### 2.1.1. Wood Specimens

Two species of wood were chosen for the study: beech (*Fagus sylvatica*) and pine sapwood (*Pinus sylvestris*). Defect-free samples were selected from the boards according to the requirements of EN 927-6 [37]. They were cut into specimens of 20 × 37 × 150 mm. 

#### 2.1.2. Titanium Dioxide

**Figure 2:**
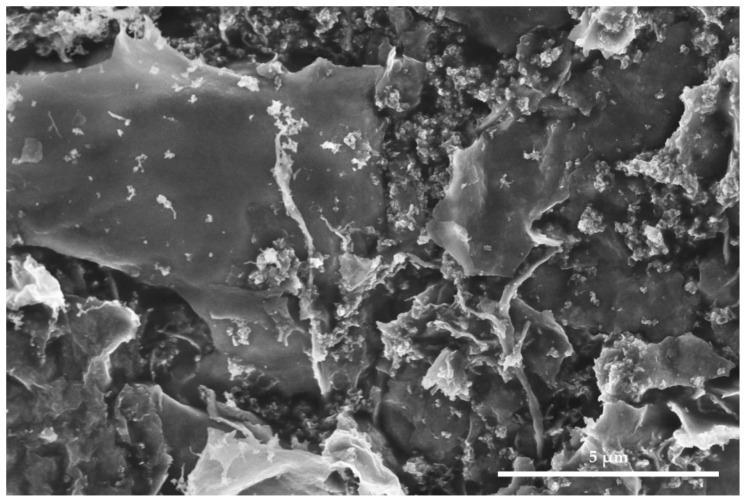
Titanium dioxide planar amorphous particle. Secondary electron photography. Magnification ×25,000 [27]. Phot. S. Svorová Pawełkowicz.

For the experiment, amorphous, non-photoactive (so by definition safe for organic matter) planar particles have been synthetized from titanium (IV) oxysulfate dihydrate (TiOSO_4_·2H_2_O) with an optimized method reported earlier [27,39]. In the final step, the solid product was annealed at 230 °C. Because of their morphology (Figure 2), these TiO_2_ planar particles could theoretically organize in a snakeskin-like hydrophobic layer. Previous research [27] showed that the morphology of the samples was not fully homogeneous and that minute quantities of crystalline phase anatase in the form of small 2–10 nm individual crystals were also present. 

#### 2.1.3. Specimens’ Preparation. Binders and Concentrations of TiO_2_ in the Mixtures 

**Figure 3:**
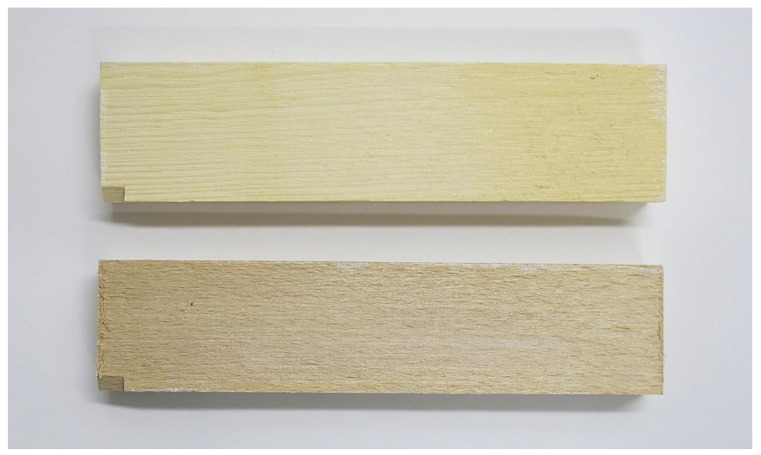
Pine wood and beech wood specimens coated with 1 wt. % of TiO_2_ acrylic dispersion. Phot. P. Svora.

Wood pieces were coated with waterborne dispersions of TiO_2,_ in different concentrations, as described in Table 1. Binders were used undiluted, in manufacturers’ concentrations. The coatings were applied by brushing, which resulted in an uneven thickness of the layers, ranging from approximately 1 µm for water as binder to up to 10 µm in the case of water glass and acrylic resin as binder. Not all the layers became transparent. After coatings application, best results in terms of coating transparency were achieved with acrylic water dispersion with 1 wt. % of TiO_2_ (Figure 3). After UV irradiation, all the coatings showed signs of degradation—the coatings became whitish or white, opaque, and were covered with micro-cracks. Reference specimens—native beech and pine wood without any coating—were prepared as well. For FTIR-ATR and TEM studies, samples with the highest TiO_2_ concentrations (3 wt. %) were chosen, as it was expected the interactions between TiO_2_ particles and the wood matter could be better articulated. 

#### 2.1.4. Accelerated Aging Test 

Accelerated aging tests were performed according to the standard EN 927-6 [37]. Exposure of wood coatings to artificial aging using fluorescent UV lamps and water was conducted on coated and native wood specimens. Each specimen was exposed to nine cycles, and each cycle corresponded to 168 h of artificial aging. One cycle consisted of two steps:

- 1st step—(24 h) Temperature 45 ± 3 °C, Water-Spray (off), UV (off)
- 2nd step—sub-cycle (A + B)—3 h
  ○ A (2.5 h) Temperature = 60 ± 3 °C, UV Irradiance = 0.89 W/m^2^ at 340 nm
  ○ (B 0.5 h) Temperature 20 ± 1 °C, Water-Spray (on), UV (off)
  Sub-cycle (A + B): 48 sub-cycles 3 h of one, i.e., together 144 h

Wood specimens have been exposed to UV radiation for a total of 1080 h.

### 2.2. Methods of Characterization

#### 2.2.1. Sample Preparation

Pure TiO_2_ planar particles were characterized with UV-VIS spectroscopy. Small samples were extracted from uncoated and coated specimens, treated and untreated with UV light for further analysis (FTIR-ATR, TEM/STEM-EELS). Prior to the extraction of samples from the specimens, XRF mapping was performed to localize the proper place for sample extraction. 

Samples for FTIR were cut out from the wood specimens.

For TEM observations, ultrathin samples were prepared. Small pieces of wood were cut out from the specimens. Samples with natural humidity (about 12 wt. %) were embedded in epoxy resin, placed in a vacuum chamber for 15 min, and left for 24 h. Once set, they were cut in the shape of a frustum pyramid. The upper surface of the frustum pyramid had the dimension of approximately 0.1 mm. The samples were cut on an ultramicrotome machine fitted with a diamond knife. The cut sections were collected from the water surface on a holey carbon copper grid. As wood is non-conductive, the samples were coated with a carbon layer; for STEM and TEM observations the carbon layer was 2–4 nm thick at both sides. This step made the samples conductive, and more stable in a vacuum and under the electron beam.

#### 2.2.2. UV-VIS Absorption Spectra of TiO_2_ Planar Particles 

UV-VIS spectroscopy was measured with a Perkin Elmer Lambda 35 spectrometer. The spectra were recorded in the transmission mode on a quartz plate. This nonstandard mode was chosen to simulate the real effect of a TiO_2_ layer covering the wood. Absorption coefficient (A_λ_ = log(I_0_/I)) was recalculated to area yield of the TiO_2_ layer. The measurement was repeated several times with various thicknesses of the layer. The final absorption value was calculated from five measurements.

#### 2.2.3. Macro X-ray Fluorescence (MAXRF)

X-ray fluorescence was recorded with an energy dispersive M6 JetStream XRF macro scanner from Bruker Nano GmbH. It utilizes an X-ray tube with a rhodium anode running at 50 kV/600 μA with a policapillary lens for beam focusing. The instrument is equipped with a 30 mm^2^ SDD (Silicon Drift Detector). The examination was conducted in air. All specimens were scanned together over the area of 610 × 345 mm^2^ in 479,140 pixels with 25 ms acquisition time in one pixel. The total live time of acquisition was 6:54 h. The presence of titanium was detected at Ti:Kα line: 4.509 keV. 

#### 2.2.4. Fourier Transform Infrared Spectroscopy with Attenuated Total Reflectance (FTIR-ATR)

FTIR spectra of the wood surface were recorded on Nicolet iS10 FT-IR spectrometer equipped with Smart iTR, using attenuated total reflectance (ATR) sampling accessory—ZnSe crystal (Thermo Fisher Scientific). The spectra were registered at an absorbance mode (A) from 4000 to 400 cm^−1^ at a spectral resolution of 4 cm^−1^, and 32 scans were used. Measurements were performed on four replicates per sample.

#### 2.2.5. Transmission Electron Microscopy (TEM) 

Transmission electron microscopy (TEM) and scanning transmission electron microscopy (STEM) were used to investigate the ultrastructure of wood. Chemical composition of the samples was studied with electron energy loss spectroscopy (EELS). TEM and STEM measurements were carried out using a FEI Tecnai TF20 X-twin microscope operated at 200 kV acceleration voltage (Thermo Fisher Scientific, Brno, Czech Republic). EELS resolution was around 0.8 eV. Additionally, a Jeol JEM-1200EX (Jeol Ltd., Tokyo, Japan) was used for TEM observations of the samples. The accelerating voltage was 120 kV.

## 3. Results and Discussion

### 3.1. UV-VIS Absorption Spectra of TiO_2_ Planar Particles

**Figure 4:**
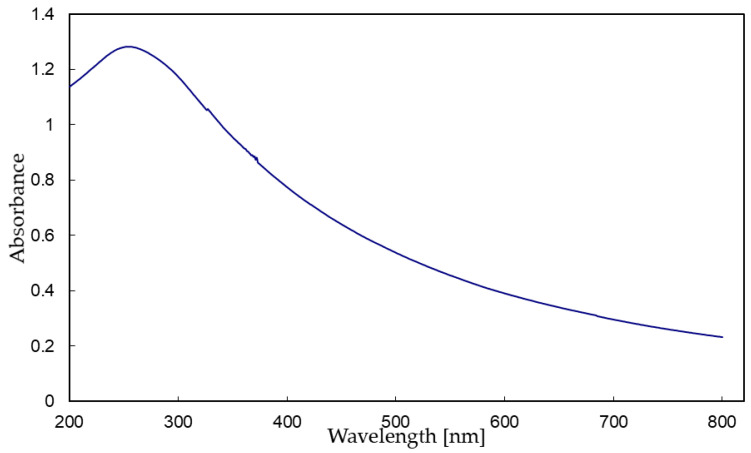
UV-VIS absorption spectra of TiO_2_ planar particles.

UV radiation is a powerful degradation agent capable of triggering delignification and crystallization of cellulose. The binding energy between atoms in cellulose and lignin macromolecules is smaller than the energy of UV light photon, that is why UV light photon is capable of breaking these bonds. TiO_2_ coating, as shown in UV-VIS spectra, absorbs some quantity of the UV and VIS radiation, but also some energy will pass through the TiO_2_ layer. Figure 4 shows UV-VIS absorption spectra of TiO_2_ planar particles in the range of 200–800 nm. The absorption maximum is at 254 nm. Absorption coefficient is recalculated to area yield of the TiO_2_ layer 1 g/m^2^. The value of the absorption coefficient is 1.28 at 254 nm for the layer 1 g/m^2^. Intensity of incident light with a wavelength of 254 nm will be attenuated 19 times after passing through the TiO_2_ layer with a “thickness” of 1 g/m^2^; in other words, only 5% of UV radiation (254 nm) will pass through the layer and reach the wood matter. 

Nano-sized TiO_2_ is reported to be quite an effective UV protective material [14,16,18,19,20,24,25,26]. The UV-VIS absorption measurement should provide information not only about UV-VIS spectra, but particularly about the UV protective function of the TiO_2_ coating. The measured absorption coefficient was therefore normalized to a TiO_2_ planar particles coating layer with an area yield (“thickness”) of 1 g of TiO_2_ spread to 1 m^2^. These measurements were conducted on pure TiO_2_ planar particles, and therefore take into account only the UV-protective function of TiO_2_.

### 3.2. X-ray Intensity Maps

**Figure 5:**
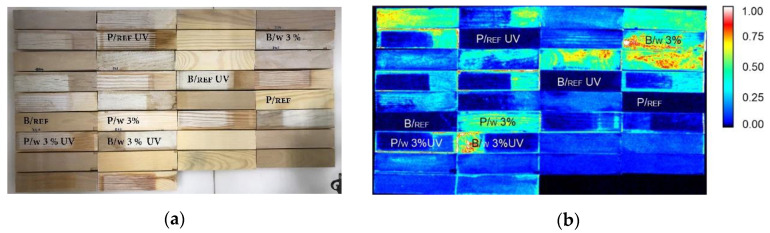
Set of specimens of beech wood (B) and pine wood (P)—uncoated (Ref) and coated with dispersions of TiO_2_ in different concentrations (3%) in water (w), water glass, and acrylic, before and after UV light irradiation: (**a**) photo in visible light; (**b**) Ti X-ray intensity map.

After artificial aging, some of the TiO_2_ coatings looked cracked and, in places, it looked as if the TiO_2_ coating had been removed from the wood surface. This was particularly the case with the specimens where water was used as binder. Besides, some of the white areas did not correspond to high TiO_2_ concentrations, but to areas of photodegradation. To correctly localize the TiO_2_ coated areas, X-ray intensity maps were developed. The maps showed exactly where the TiO_2_ coating was still present in wood specimens (Figure 5) after exposition to artificial ageing. 

### 3.3. Fourier Transform Infrared Spectroscopy with Attenuated Total Reflectance (FTIR-ATR)

When studying photodegradation of wood, bands at 800–1800 cm^−1^ are considered as the fingerprint region [40,41]. According to Cogulet et al. [40], in the first step of the photodegradation process, yellowing of wood matter is directly linked to lignin photodegradation, while the appearance of silver patina marking the final step of photodegradation is related to generation of carbonyl compounds observable at 1615 cm^−1^ in the FTIR spectra. Lignin is the chemical component that is the most sensitive to UV light [42]. As stated by Cogulet et al., hemicelluloses are more sensitive to photodegradation than cellulose. Bands at 1420–1430 cm^−1^ are associated with the crystalline structure of cellulose [43]. Table 2 lists the bands described in the literature and observed in the studied samples.

**Figure 6:**
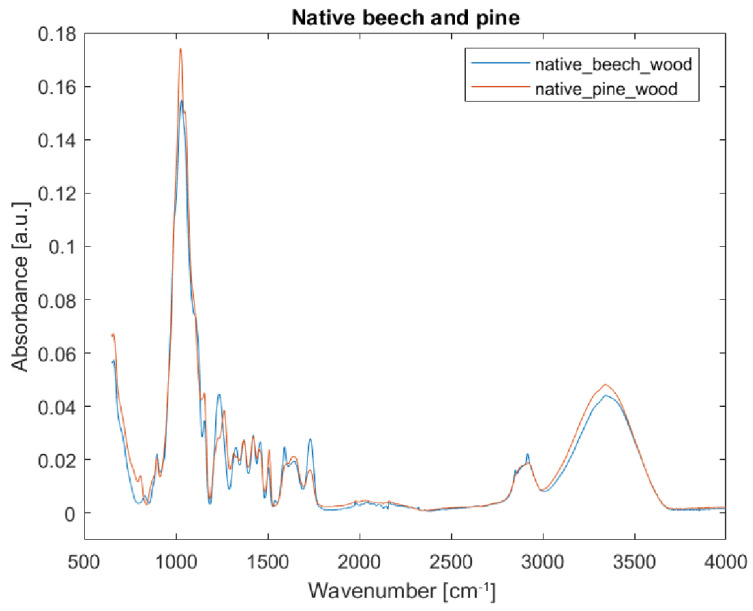
FTIR-ATR spectra of native (blue) beech and (red) pine wood reference samples.

Chemical composition of beech (*Fagus sylvatica*) and pine sapwood (*Pinus sylvestris*) is not identical, but similar. The spectra of native pine wood differ from those of beech wood (see Figure 6) by the presence of bands at 1265 and 806 cm^−1^, absence of bands at 830 and 2850 cm^−1^, and a decrease in band intensity at 1608 cm^−1^ in pine wood (as compared to beech). Pine wood is a coniferous wood composed of cellulose (40.3%), hemicelluloses (28.7%), and mannan lignin (15–36%). Beech wood is a deciduous wood composed of cellulose (39.2%), hemicelluloses (35.3%), and lignin (20.9%) [47].

#### 3.3.1. Fourier Transform Infrared Spectroscopy of Specimens with Water as Binder 

**Figure 7:**
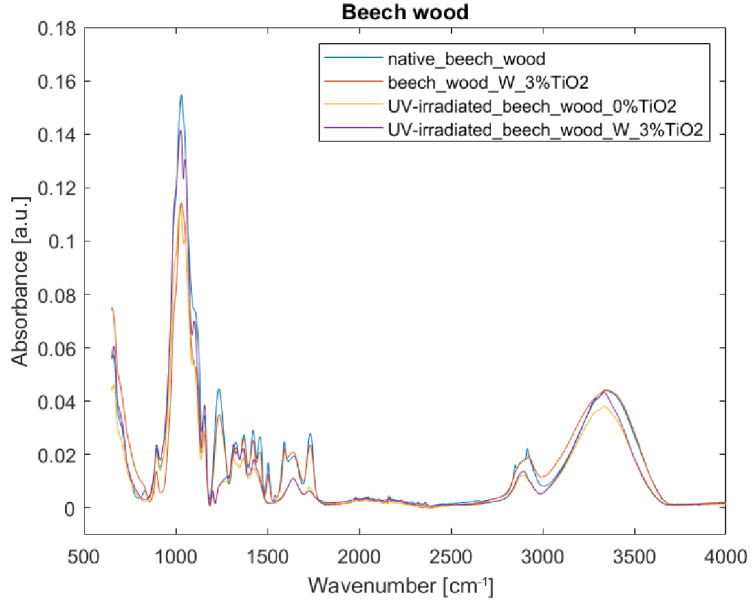
FTIR-ATR spectra of beech wood samples: (blue) reference sample of native beech wood, (red) beech wood with 3% TiO_2_ coating with water as binder, (yellow) UV-irradiated beech wood without TiO_2_ coating, and (purple) UV-irradiated beech wood with 3% TiO_2_ coating with water as binder.

**Figure 8:**
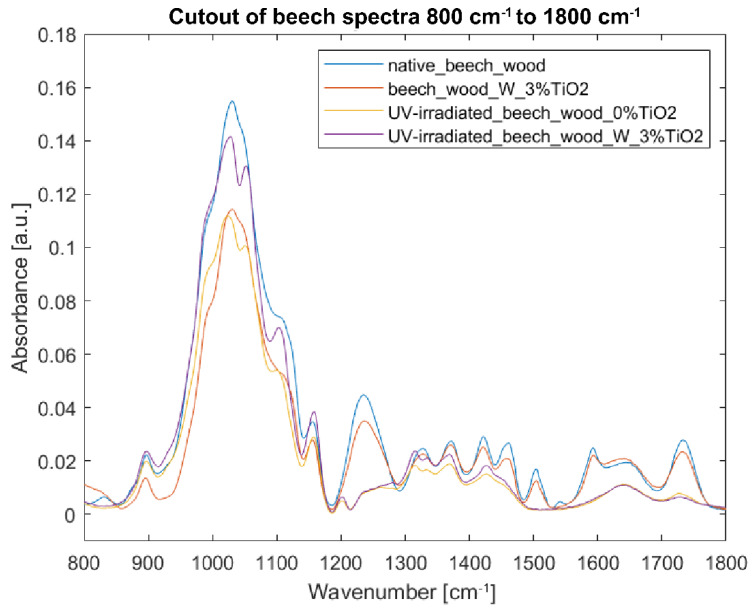
FTIR-ATR spectra of beech wood samples in the fingerprint region 800–1800 cm^−1^: (blue) reference sample of native beech wood, (red) beech wood with 3% TiO_2_ coating with water as binder, (yellow) UV-irradiated beech wood without TiO_2_ coating, and (purple) UV-irradiated beech wood with 3% TiO_2_ coating with water as binder.

Figure 7 and Figure 8 show that TiO_2_ coating is not well-visible in the FTIR-ATR spectra. The wide band at 400–800 cm^−1^ attributed to Ti-O-Ti stretching vibration [18,24] overlaps with the band present in the wood spectra. Generally, bands from an uncoated sample are stronger than those from a coated one, especially at 1031 and 1045 cm^−1^. The exception to the rule are the bands at 1641, and between 3000 and 3500 cm^−1^—in these cases, intensities of absorptions are slightly increased in comparison to those of an uncoated sample, and can be assigned to water absorption and hydrogen bonds development [43]. Figure 8 shows substantial changes in the molecular structure of wood without any coating irradiated with UV light. Intensities in the spectrum of the irradiated sample decreased (e.g., 1024, 1461, and 1645 cm^−1^), and some bands seem not to be present in the spectrum anymore (e.g., 831, 1236, 1327, 1504, 1540, and 1592 cm^−1^). This should be attributed to the loss of water (1645 cm^−1^), delignification (see wavelength assignments for 1236, 1461, and 1504 cm^−1^ in Table 2), and decrease in cellulose crystallinity (see wavelength assignments for 1024 and 1327 cm^−1^ in Table 2). After irradiation of the uncoated sample, due to the destruction of some bonds under UV light, some bands, which, in native wood, were hidden in envelope curves, became more visible, i.e., bands at 1051 (C-O stretching vibration in cellulose and hemicelluloses), 1201 (CH_2_ and O-H deformation in cellulose), 1315 (C-H vibration in cellulose and C1-O vibration in syringyl), and 1335 cm^−1^ (CH_2_ wagging and O-H deformation in cellulose). Additionally, in the TiO_2_-coated sample treated with UV light, a band appeared in the spectrum at 2895 rather than at 2850 and at 2915 cm^−1^, and a shoulder at 3334 cm^−1^. The increase in band height at 897 and 1155 cm^−1^ should be assigned to C1-H and C-O-C vibration in cellulose and hemicelluloses respectively. Figure 7 and Figure 8 show reduced absorbance values for the uncoated sample irradiated with UV light as compared to the coated one, thus proving that the TiO_2_ layer, to a small extent, effectively protects the wood specimen. Limited effectiveness of TiO_2_-based UV-protective layers is consistent with the previous studies. Unfortunately, most of the layers do not withstand weathering—depolymerized wood components leak through the micro-cracks [22].

**Figure 9:**
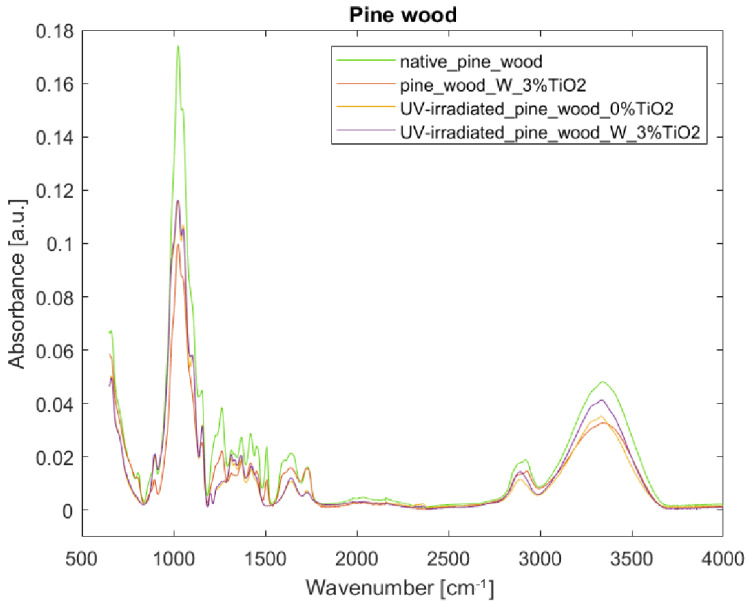
FTIR-ATR spectra of pine wood samples: (green) reference sample of native pine wood, (red) pine wood with 3% TiO_2_ coating with water as binder, (yellow) UV-irradiated pine wood without TiO_2_ coating, and (purple) UV-irradiated pine wood with 3% TiO_2_ coating with water as binder.

**Figure 10:**
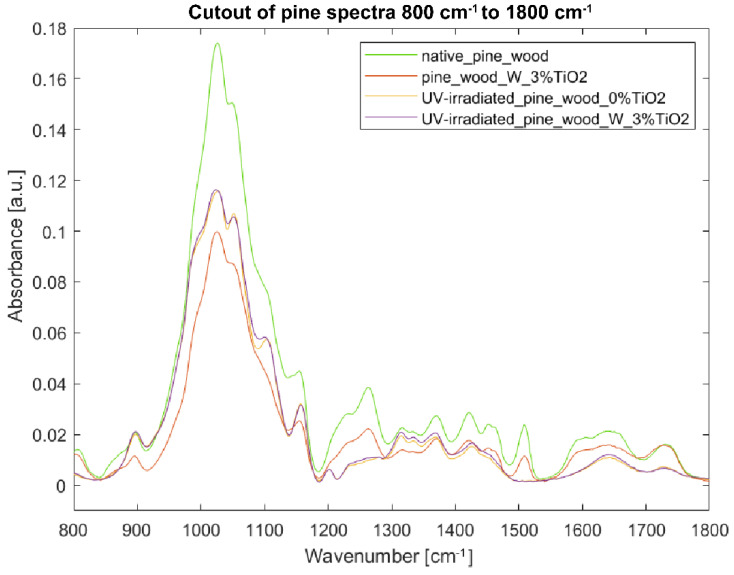
FTIR-ATR spectra of pine wood samples in the fingerprint region 800–1800 cm^−1^: (green) reference sample of native pine wood, (red) pine wood with 3% TiO_2_ coating with water as binder, (yellow) UV-irradiated pine wood without TiO_2_ coating, and (purple) UV-irradiated pine wood with 3% TiO_2_ coating with water as binder.

Figure 9 and Figure 10 show pine wood’s reaction to UV light, with and without TiO_2_ coating. Like in the case of beech wood, the TiO_2_ coating is not well-visible in the spectrum. There is a clear decrease in band intensities in the spectrum of the sample coated with TiO_2_ as compared to the uncoated one, except for the band at 1730 cm^−1^ assigned to C=O stretch of acetyl or carboxylic acid in hemicelluloses. Most of the intensities in the spectrum of the uncoated UV-irradiated sample decreased compared to the sample of native pine wood (e.g., 1028, 1051, 1103, 1157, 1230, 1262, 1315, 1335, 1369, 1419, 1452, 1643, 1730, 2883, 3282, and 3344 cm^−1^). Some bands are no longer present in the spectrum (e.g., bands at 806, 1508, 1600, and 2920 cm^−1^), which shows the bonds’ degradation induced by UV light. Some bands, present as minute shoulders in the spectra of samples untreated with UV light, became more visible in the spectra of the irradiated samples; this is the case of bands at 1201 cm^−1^ (CH_2_ and O-H deformation in cellulose [46]), and 2893 cm^−1^. The spectra of uncoated and coated specimens irradiated with UV light show similar trends; at certain wavenumbers, they overlap (see 806–1201 cm^−1^), or the spectrum of the uncoated sample shows slightly reduced absorbance (see 1317–1639 cm^−1^). In the range 2800–3500 cm^−1^, there is a clear decrease in band intensity in the spectrum from the uncoated sample as compared to the coated one. In the case of pine wood, the protective effect of TiO_2_ coating is not so clearly visible as in the case of beech wood samples.

#### 3.3.2. Fourier Transform Infrared Spectroscopy of Specimens with Acrylic Resin as Binder 

**Figure 11:**
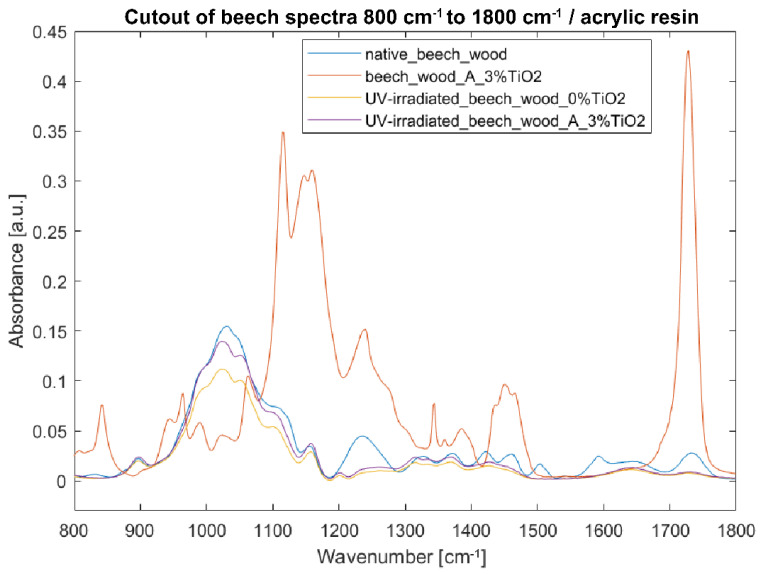
FTIR-ATR spectra of beech wood samples in the fingerprint region 800–1800 cm^−1^: (blue) reference sample of native beech wood, (red) beech wood with 3% TiO_2_ acrylic coating, (yellow) UV-irradiated beech wood without TiO_2_ coating, and (purple) UV-irradiated beech wood with 3% TiO_2_ acrylic coating.

**Figure 12:**
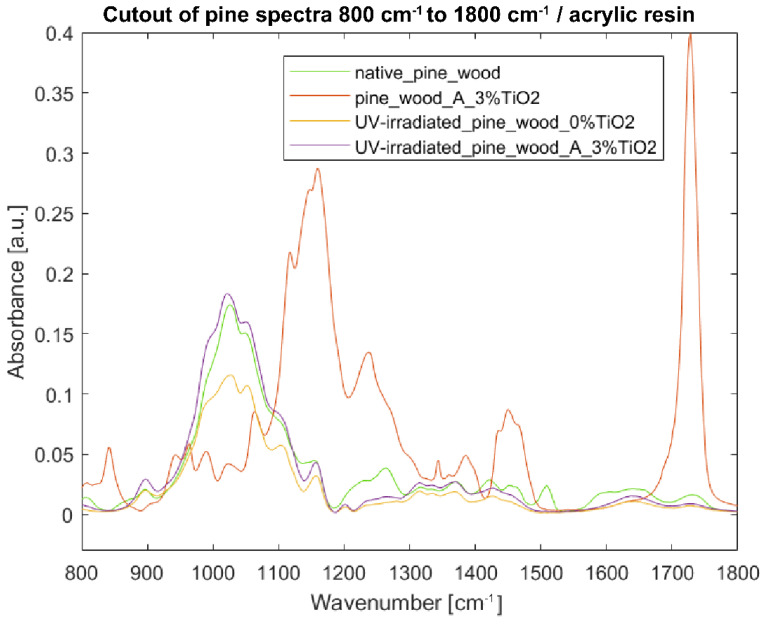
FTIR-ATR spectra of pine wood samples in the fingerprint region 800–1800 cm^−1^: (green) reference sample of native pine wood, (red) pine wood with 3% TiO_2_ acrylic coating, (yellow) UV-irradiated pine wood without TiO_2_ coating, and (purple) UV-irradiated pine wood with 3% TiO_2_ acrylic coating.

Spectra acquired from specimens coated with TiO_2_ acrylic dispersion are much more complicated, as the influence of the synthetic polymer is visible. Therefore, only the fingerprint region (bands at 800–1800 cm^−1^) was analyzed (Figure 11 and Figure 12).

In the case of beech wood, the intensities in the spectra of the irradiated samples (coated and uncoated) decreased compared to the native wood’s spectrum (Figure 11). Clear signs of delignification were observed with the decrease in intensities at 1236, 1460, and 1504 cm^−1^. The most significant decrease in intensities was observed at 1030, 1047, and 1100 cm^−1^ assigned to C=O, C-O, and O-H stretching vibration in cellulose, hemicelluloses, and lignin [40,44,45,46]. In this area, the bands of the coated sample are much higher than of those of the uncoated one, proving the efficiency of the coating. In the region 1200–1800 cm^−1^, the spectra of the coated and uncoated samples overlap. 

In the case of pine wood, the intensities of the coated irradiated sample are higher than those of the uncoated irradiated sample, and both are lower in the range of 1200–1800 cm^−1^ than the ones of native pine wood (Figure 12). Interestingly, the bands at 1022, 1050, and 1103 cm^−1^ in the spectrum from the coated irradiated sample are stronger or equal to the intensities observed for native pine wood (at 1026, 1049, and 1103 cm^−1^).

#### 3.3.3. Fourier Transform Infrared Spectroscopy of Specimens with Water Glass as Binder 

**Figure 13:**
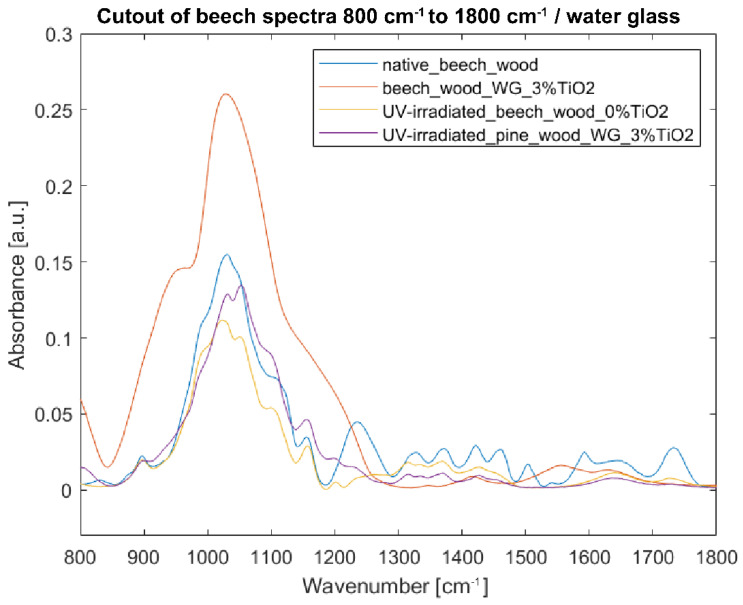
FTIR-ATR spectra of beech wood samples in the fingerprint region 800–1800 cm^−1^: (blue) reference sample of native beech wood, (red) beech wood with 3% TiO_2_ water glass coating, (yellow) UV-irradiated beech wood without TiO_2_ coating, and (purple) UV-irradiated beech wood with 3% TiO_2_ water glass coating.

**Figure 14:**
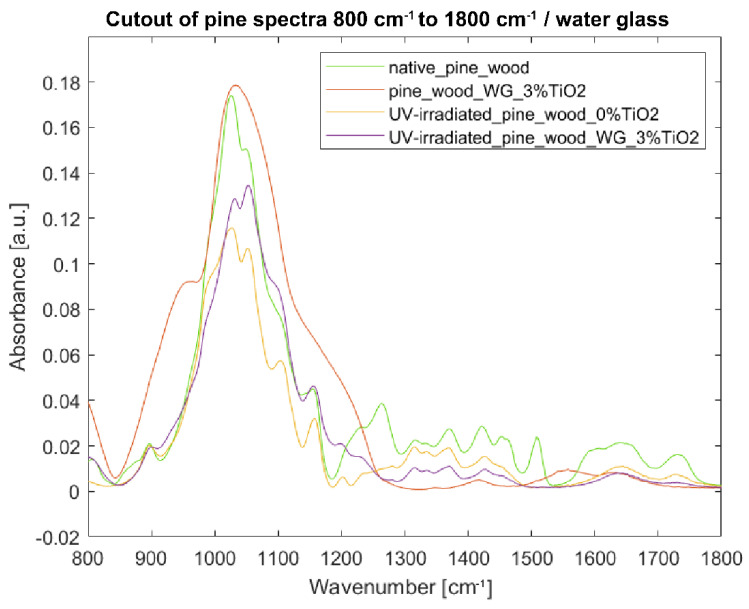
FTIR-ATR spectra of pine wood samples in the fingerprint region 800–1800 cm^−1^: (green) reference sample of native pine wood, (red) pine wood with 3% TiO_2_ water glass coating, (yellow) UV-irradiated pine wood without TiO_2_ coating, and (purple) UV-irradiated pine wood with 3% water glass TiO_2_ coating.

Spectra of samples with water glass as binder are the most difficult to analyze as the intensities of water glass dominate over native wood in the range of 800–1250 cm^−1^, both in the case of beech and pine wood (Figure 13 and Figure 14). Spectra of coated irradiated samples of beech and pine wood copy the trend of the not-irradiated coated samples—especially in the range of 800–1250 cm^−1^. In the range of 1300–1800 cm^−1^, spectra of coated irradiated samples start to copy the trend of uncoated irradiated samples. Nevertheless, the intensities of coated samples are much lower than those of the uncoated ones—which, given the impact of the water glass binder, is not necessarily related with depolymerization of wood but rather reflects the vibrations coming from the binder.

#### 3.3.4. Photodegradation Parameters 

Cogulet et al. [40] adopted a ratio of lignin band at 1510 cm^−1^ to the carbohydrate band at 1375 cm^−1^ (I_1510_/I_1375_) for the observation of the delignification rate caused by UV irradiation and a ratio of bands at 1316 to 1335 cm^−1^ (I_1316_/I_1335_) to monitor the crystallinity of cellulose. 

The observed changes in the molecular structure of wood exposed to UV irradiation are in compliance with the literature [40,42], especially as far as delignification is concerned. Delignification was proved on the basis of calculated ratio I_1510_/I_1375_ (Table 3). The lower the ratio, the more depolymerized the lignin. It was shown that a TiO_2_ coating with all the tested binders did not prevent lignin from depolymerization in either beech or pine wood. In the case of pine wood, the value of I_1510_/I_1375_ obtained for the sample coated with TiO_2_ and water as binder was even lower than for the uncoated one. Higher indexes calculated for the samples coated with TiO_2_ dispersions in acrylic resin or water glass do not necessarily mean the depolymerization rate is lower, as the results are strongly influenced by the presence of binders. 

Cogulet et al. [40] reported an increase in cellulose crystallinity in the first step of the woods’ photodegradation, followed by a decrease in cellulose crystallinity and depolymerization. They proposed an index based on the ratio of bands at 1316 and 1335 cm^−1^ (I_1316_/I_1335_)—the higher the index, the higher the crystallinity of cellulose. The results of this study are not fully consistent with those of Cogulet et al. The index slightly increased in the case of irradiated pine wood (both coated and uncoated). As far as beech wood is concerned, the index of the irradiated uncoated sample decreased, but it increased for irradiated coated samples. No difference in I_1316_/I_1335_ was observed in the results from samples coated with acrylic resin (irradiated and not irradiated). 

A simultaneous decrease in band intensities at 1508 and 1730 cm^−1^ and the lack of a band at 1615 cm^−1^ is contrary to Cogulet et al. [40] and Muller et al. [48], who observed a decrease in the range of 1510–1600 cm^−1^ along with an increase in bands at 1615 and 1700–1650 cm^−1^, respectively, and interpreted it as formation of conjugated carbonyl groups accompanying the decay of the aromatic structure of lignin. 

In all the samples irradiated with UV light, a loss of water molecules was observed. However, in the case of samples with a TiO_2_ coating, the decrease in relevant intensities was slightly less important. This phenomenon could be explained by referring to the properties of TiO_2_ to chemisorb water on its surface under UV light [11].

### 3.4. Transmission Electron Microscopic (TEM) Study of Beech and Pine Wood’s Cell Walls Ultrastructure 

**Figure 15:**
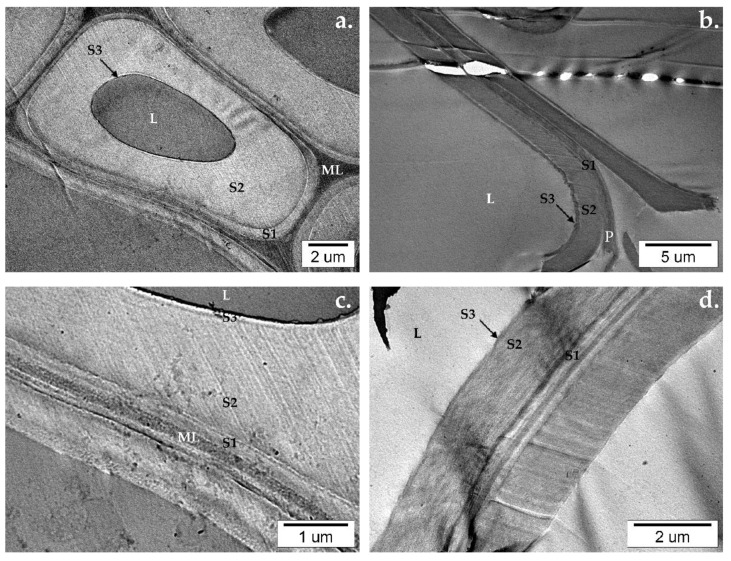
TEM micrographs of (**a**,**c**) native beech and (**b**,**d**) pine wood’s cell wall ultrastructure.

TEM micrographs show how the molecular and chemical changes visible in the FTIR-ATR spectra result in changes on the level of the ultrastructure of wood’s cell walls. In the reference samples of native beech and pine wood untreated with UV light, the wood’s cell walls ultrastructure remains intact. It is possible to discern the constituents of the wood cell: middle lamella (ML), primary wall (P), secondary walls (S1, S2, S3), and lumen (L) (Figure 15).

**Figure 16:**
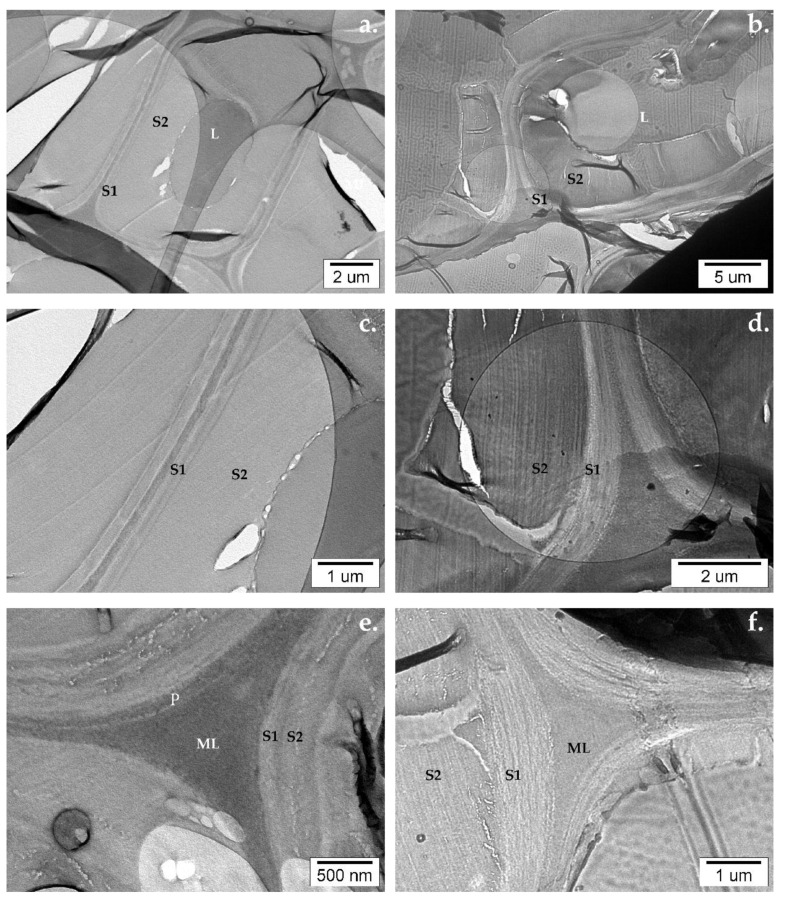
TEM micrographs of uncoated sample irradiated with UV light: (**a**,**c**,**e**) beech and (**b**,**d**,**f**) pine wood’s cell wall ultrastructure.

Micrographs from samples irradiated with UV light and uncoated with TiO_2_ show significant changes occurring especially in the middle layer of the secondary wall (S2). Both in beech and pine wood, numerous fissures appear, mostly perpendicular to the S1 layer. The fissures start in the area of the S3 layer. Small perforations forming fissures are visible in the S2 layer of the beech (Figure 16e) and pine wood cells (Figure 16d,f). 

**Figure 17:**
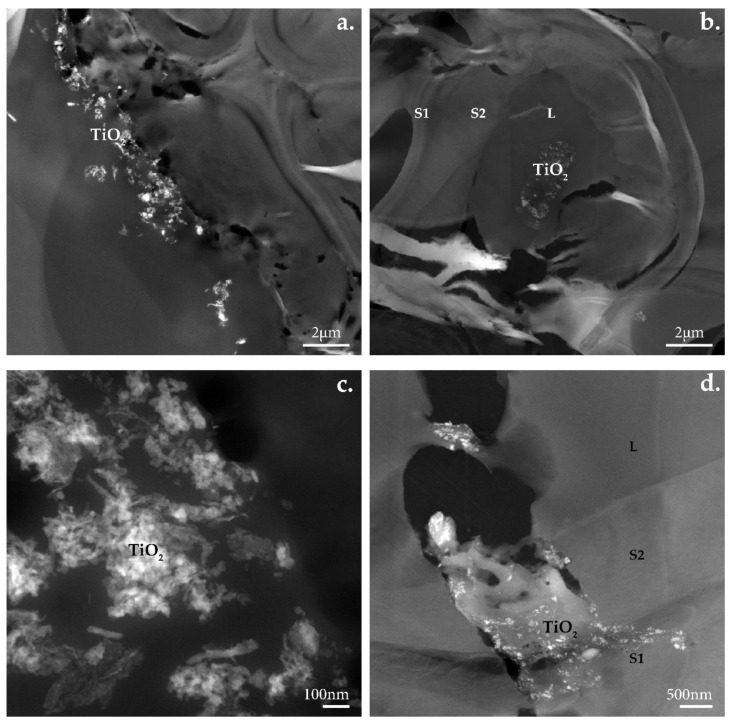
TEM micrographs of TiO_2_ coated samples before irradiation: (**a**,**c**) beech and (**b**,**d**) pine wood’s cell wall ultrastructure.

The presence of TiO_2_ in the samples of coated wood was confirmed with EELS. Fragile, several-micron-large planar TiO_2_ particles, when mixed with water to create a coating, broke into smaller planar particles of hundreds of nanometers or minute individual particles of 25–30 nm as seen in Figure 17. In only a few places, the TiO_2_ coating stayed on the wood’s surface (Figure 17a). In some cases, clusters of TiO_2_ particles were found in the lumen (Figure 17b) or in the wood’s structure (Figure 17d). Fissures and holes appeared in the vicinity of TiO_2_ clusters (Figure 17d). 

**Figure 18:**
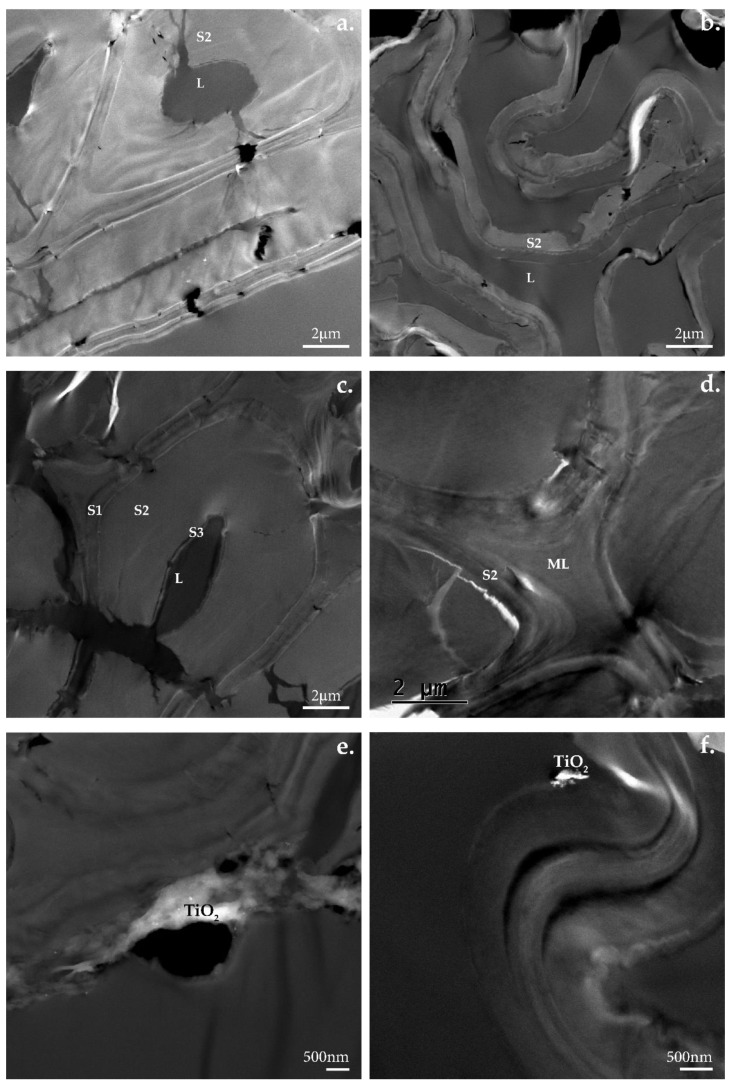
TEM micrographs of TiO_2_ coated samples before irradiation: (**a**,**c**,**e**) beech and (**b**,**d**,**f**) pine wood’s cell wall ultrastructure.

**Figure 19:**
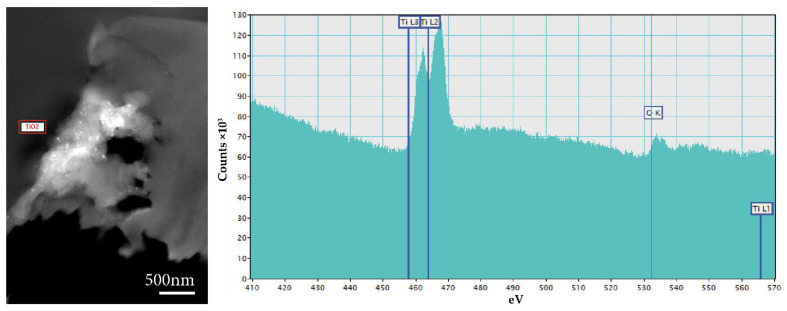
TEM micrograph of TiO_2_ cluster in the coated and irradiated sample of beech wood (**left**), and EELS spectrum of TiO_2_ (**right**).

TEM observations of samples extracted from specimens coated with TiO_2_ and exposed to UV light irradiation confirmed the observations made after the FTIR-ATR analysis of the samples. The wood’s cell wall ultrastructure showed evident signs of degradation. Fissures were present, especially in the S2 layer perpendicular to the S1 layer (Figure 18a). In the case of beech wood, in some cells, the S3 layer detached from the S2 layer (Figure 18b). In the case of pine wood, the wood’s cell walls underwent strong deformation (Figure 18b,f). The S2 layer shrank and the walls started to curl. No evident changes were observed around the clusters of TiO_2_ in the case of pine wood. As for beech wood, perforations and fissures appeared in the vicinity of the TiO_2_ clusters (Figure 19). 

## 4. Conclusions

UV-VIS spectroscopy confirmed that the planar TiO_2_ particles absorb UV and VIS radiation, with the absorption maximum peak at 254 nm, and that some energy passes through the TiO_2_ layer. Comparison of FTIR-ATR spectra demonstrated the relatively low effectiveness of a TiO_2_ protection layer against UV radiation for beech (*Fagus sylvatica*) and pine (*Pinus sylvestris*) wood. The coating was slightly more efficient in the case of beech wood than pine wood. The expected organization of TiO_2_ planar particles in a snakeskin-like hydrophobic layer has not been observed. On the contrary, large particles broke into smaller ones and moved into the wood’s structure. 

The FTIR-ATR spectra demonstrated that depolymerization of lignin in UV-irradiated samples was slightly more noticeable in the case of pine wood coated with TiO_2_ than in uncoated pine wood. As far as beech wood is concerned, the TiO_2_ coating had no effect on delignification. Nevertheless, TEM micrographs revealed fissures and holes around TiO_2_ clusters in the case of UV-irradiated beech wood, proving an interaction between TiO_2_ particles and wood matter. Micrographs from samples irradiated with UV light, uncoated and coated with TiO_2_, show significant signs of degradation in the wood cell ultrastructure.

## Figures and Tables

**Table 1:** Binders and concentrations of TiO_2_ in the mixtures.

Binder	Concentrations (wt. %)		
H_2_O	0.5	1.5	3
Potassium water glass (MM 1.6 by Vodnisklo a.s.)	0.5	1.5	3
Acrylic water dispersion (Primal^®^ SF016 by Rohm&Haas)	0.5	1.5	3

**Table 2:** Assignment of IR characteristic bands for wood degradation after Cogulet [40], Bari [44], Ozgenc [41], Dirckx [45], and Pandey [46].

Literature Band (cm^−1^)	Observed Band (cm^−1^)	Compound or Chemical Group
806, 812, 813	806	C-C deformation and stretching vibration in mannans [45]
-	827–831	Observed in beech wood only
895–897	893–897	C-H deformation in cellulose [40,44] C1-H group vibration in cellulose and hemicelluloses [41,46]
1026, 1029, 1030, 1031, 1033	1024, 1028, 1030–1032	C-O stretching vibration in cellulose [46], hemicelluloses [40,41], C=O stretching vibration in cellulose, hemicelluloses, and lignin [44] C-O of primary alcohol, C-H in guaiacyl [46]
1050–51, 1052, 1059	1045, 1051, 1053	C-O stretching vibration in cellulose [46] and hemicelluloses [40]
1097	1099	Aromatic C-H in-plane deformation and C=O stretch O-H association band in cellulose and hemicelluloses [44]
1104, 1109, 1115	1101, 1103	Aromatic skeletal vibration and C-O stretch [40] C-O and O-H stretching vibration [45]
1134, 1152, 1155, 1156, 1157, 1160, 1163, 1165	1155–1157	C-O-C vibration in cellulose and hemicelluloses [40,41,44] and lignin [45]
1200, 1208	1201	O-H deformation in (1200 cm^−1^) cellulose [46] and CH_2_ and O-H deformation (1208 cm^−1^) hemicelluloses [45]
1222, 1230, 1233, 1234	1230–1236	C=O stretching vibrations in lignin, acetyl and carboxyl vibrations in xylans [44] C-O stretch in lignin [45] and xylan [41] Syringyl ring [41]
1252, 1260, 1265, 1266, 1267, 1268, 1280	1262–1265	Guaiacyl ring breathing [41] C-O stretch in lignin and mannans [40,41,45] C-O linkage in guaiacyl aromatic methoxyl groups [41]
1309, 1313, 1314, 1316–1326, 1318	1315–1317, 1327–1329	C-H vibration in cellulose [40,41] CH_2_ wagging in cellulose [46] C1-O vibration in syringyl derivatives [41,44] CH_2_ and O-H deformations in cellulose and hemicelluloses [45]
1330, 1333, 1335	1335 shoulder	CH_2_ wagging [45] and O-H deformation in cellulose [45,46]
1367, 1368–1372, 1375	1369–1371	C-H deformation in cellulose [46] and hemicelluloses [40,41,44] and lignin [45]
1408, 1417, 1419, 1421, 1422–1424, 1425, 1430	1419–1421	C-H asymmetric deformation in –OCH_3_ [44,46] Aromatic skeletal vibrations [44] C-H deformation in lignin [46] and carbohydrates [41,44] CH_2_ and CH_3_ deformation in cellulose, lignin and hemicelluloses [45]
1451-56, 1452, 1455, 1458, 1460, 1462, 1463	1452–1462	C-H deformation in lignin [40,46] and carbohydrates [41] CH_2_ deformation vibrations in lignin and xylans [44] CH_2_ and CH_3_ deformation in cellulose, lignin and xylans [45]
1502, 1504, 1506–1509, 1510	1504–1508	Aromatic skeletal vibration in lignin [40,41,44,46] C=C stretching of the aromatic ring in guaiacyl [44,45]
-	1541	C=O stretching vibration
1592, 1593, 1595, 1598, 1605, 1606, 1610	1592	C=C stretching of the aromatic ring in syringyl [44,45] Aromatic skeletal vibrations and C=O stretching [44,46] Conjugated C-O stretching [41]
1615	-	C=O stretching conjugated to double bond [40]
1635, 1640	1639–1645	H-O-H deformation vibration of absorbed water [44,46] C=O stretching in lignin [44,45] and in cellulose [46]
1720, 1730–1732, 1734	1730–1732	C=O stretch of acetyl or carboxylic acid in hemicelluloses [40] C=O stretching in xylans (unconjugated) [41,44]
2800–3000	2850, 2883, 2893, 2895, 2916–2918, 2920, 2924–2928	C-H stretching [44,45]
3300–4000	3282–3304, 3334, 3342–3346	Strong broad O-H stretching absorption band [45]

**Table 3:** Photodegradation parameters: rate of delignification (ratio of band at 1510 to 1375 cm^−1^), crystallinity of cellulose (ratio of band at 1316 to 1335 cm^−1^).

Sample	I_1510_/I_1375_	I_1316_/I_1335_
native_beech_wood	0.62	0.94
UV-irradated_beech_wood_0%TiO_2_	0.09	0.87
beech_wood_3%TiO_2_ in water	0.49	0.93
UV-irradiated_beech_wood_3%TiO_2_ in water	0.09	1.43
beech_wood_3%TiO_2_ in acrylic	0.14	1.09
UV-irradiated_beech_wood_3%TiO_2_ in acrylic	0.09	1.09
beech_wood_3%TiO_2_ in water glass	3.32	0.79
UV-irradiated_beech_wood_3%TiO_2_ in water glass	0.17	1.12
		
native_pine_wood	0.87	1.07
UV-irradiated_pine_wood_0%TiO_2_	0.08	1.12
pine_wood_3%TiO_2_ in water	0.63	1.05
UV-irradiated_pine_wood_3%TiO_2_ in water	0.07	1.11
pine_wood_3%TiO_2_ in acrylic	0.14	1.16
UV-irradiated_pine_wood_3%TiO_2_ in acrylic	0.10	1.08
pine_wood_3%TiO_2_ in water glass	3.49	0.87
UV-irradiated_pine_wood_3%TiO_2_ in water glass	0.15	1.12

## Data Availability

Data will be made available on request.
